# Psychological Stress, Family Environment, and Constipation in Japanese Children: The Toyama Birth Cohort Study

**DOI:** 10.2188/jea.JE20180016

**Published:** 2019-06-05

**Authors:** Masaaki Yamada, Michikazu Sekine, Takashi Tatsuse

**Affiliations:** Department of Epidemiology and Health Policy, Graduate School of Medicine and Pharmaceutical Sciences, University of Toyama, Toyama, Japan

**Keywords:** constipation, family, psychological stress, sex difference, the Toyama Birth Cohort Study

## Abstract

**Background:**

Childhood constipation is prevalent and negatively affects quality of life. Although psychological stress and family environment have been identified as risk factors, few epidemiological studies have examined this issue. We aimed to clarify associations of psychological stress and family environment with childhood constipation in a large-scale epidemiological study.

**Methods:**

In total, 7,998 children aged 9–10 years from the Toyama Birth Cohort Study completed questionnaires. Constipation was defined as bowel movements “less frequently than once every 2 days”. Children’s lifestyles, including food frequency, psychological stress, family environment, frequency of irritability, unwillingness to attend school, and frequency of interaction with their parents, were analyzed via multivariate logistic regression analysis. Parental employment status and presence at dinner were also examined.

**Results:**

In total, 312 children (3.9%) experienced constipation. Girls were more likely to experience constipation than boys (5.1% vs 2.8%). In addition, constipation was significantly associated with girl (odds ratio [OR] 1.97; 95% confidence interval [CI], 1.55–2.51), physical inactivity (OR 1.41; 95% CI, 1.01–1.95), overweight (OR 0.58; 95% CI, 0.40–0.85), infrequent fruit (OR 1.94; 95% CI, 1.42–2.66) and vegetable (OR 1.46; 95% CI, 1.03–2.05) consumption, frequent irritability (OR 1.76; 95% CI, 1.24–2.50), unwillingness to attend school (OR 1.66; 95% CI, 1.13–2.43), and infrequent interaction with parents (OR 1.48; 95% CI, 1.06–2.07). Children whose parents were absent at dinner were more likely to experience constipation compared to those whose parents were present at dinner; however, this differences were not statistically significant.

**Conclusion:**

Psychological stress and infrequent interaction with parents were as strongly associated with childhood constipation as conventional risk factors. Psychological stress and family environment should be more prioritized in caring childhood constipation.

## INTRODUCTION

Constipation is a globally prevalent gastrointestinal disorder. The prevalence of childhood constipation has been reported to range from 0.7% to 29.6%.^1^^,^^2^ Moreover, in many cases it is not self-limiting, and despite treatment with laxatives, prognoses are not always good.^3^^,^^4^ For instance, Bongers et al showed that 25% of children with constipation remained symptomatic at the age of approximately 20 years.^3^ Furthermore, constipation negatively affects both individuals and society. Belsey et al reported that constipation decreased individuals’ quality of life.^5^ In addition, constipation contributes to 2.5 million physician visits annually and generates considerable healthcare costs for American society.^1^ Taking into account its effects on individuals and society, childhood constipation should be considered a serious concern, and it is important to identify modifiable factors in everyday life.

Several risk factors, such as insufficient fiber intake, genetic predisposition, volitional stool retention, physical inactivity, low socioeconomic status, and psychological status, have been identified for constipation.^1^^,^^2^ However, factors related to children’s psychological status, such stress and personality, have not been examined comprehensively in a large population. Ozokutan et al reported no differences in behavioral or emotional problems between 32 and 30 children who were and were not constipated, respectively.^6^ In contrast, Devanarayana et al reported a significant association between emotional stress and bowel habits in 2,273 children in Sri Lanka.^7^ This discrepancy could have occurred because most studies involving psychological status have included small- or medium-sized samples.^8^^,^^9^ In addition, factors related to family environment, such as parent-child relationships and parental presence at dinner, were reported as predisposing factors for childhood constipation.^9^^,^^10^ For example, Tam et al showed that children in Hong Kong whose parents were rarely present at dinner were more likely to have constipation compared to children whose parents were regularly present at dinner.^10^ According to the previous literature, mothers’ employment status affected their children’s weight and led to undesirable nutrition patterns.^11^^,^^12^ Gaina et al showed that children of mothers in full-time employment were more likely to snack and skip dinner, and those whose mothers were in part-time employment ate larger portions compared to those with unemployed mothers.^11^^,^^12^ Recently, women’s participation in the labor force has increased in Japan,^13^ and the number of working women increased from 26.5 million in 2006 to 28.0 million in 2016. Therefore, we identified the need to assess family environmental factors and hypothesized that these factors would affect children’s constipation via psychological and nutritional pathways.

The aims of our study were to clarify the prevalence of constipation in Japanese children and estimate the effects of psychological stress and family environment by comprehensively comparing these factors with conventional risk factors for constipation, such as dietary intake and physical activity, in a large epidemiological study. To the best of our knowledge, this is the largest epidemiological study examining the association of childhood constipation with lifestyle, psychological stress, and family environment.

## METHODS

### Participants and the Toyama Birth Cohort Study

The participants were children from the Toyama Birth Cohort Study, a prospective, longitudinal survey examining lifestyle and health in 10,438 children born in Toyama Prefecture, Japan, between April 2, 1989 and April 1, 1990. The cohort of schoolchildren had been evaluated via questionnaire every 3 years from Phase 1 in 1992 to Phase 5 in 2005. The overall purpose of the Toyama Birth Cohort Study was to clarify the effects of lifestyle factors and family environment on children’s health, and the details have been published elsewhere.^11^^,^^12^^,^^14^^–^^16^ We conducted a cross-sectional study and analyzed data from Phase 3 of the Toyama Birth Cohort Study because the questionnaire used in this phase included items pertaining to food consumption frequency, in addition to lifestyle and family environment. The data were collected in June and July 1999, when the cohort of children were in Grade 4 at elementary school and aged 9–10 years. The prefecture education authorities approved the content and ethical aspects of the current study, and the Toyama Birth Cohort Study was approved by the institutional review board at Toyama Medical and Pharmaceutical University. Written informed consent was obtained from the participants’ parents, and participation was voluntary.

### Questionnaire

A self-administered structured questionnaire was distributed to school children and included items pertaining to four main areas: lifestyle, including physical activity and food consumption frequency; psychological stress; family environment; and health status, including anthropometry data and bowel movements. Children responded to questions pertaining to their lifestyles, psychological status, child-parent interaction at home, and bowel movements, while their parents provided information regarding their employment status, their children’s food consumption frequency, and anthropometry data by referring to their school health checks. The lifestyle items included skipping breakfast (yes or no), physical activity (very often, often, or rare), and hours spent watching TV on weekdays (<2 h, 2 to <3 h, or ≥3 h). We divided sleep duration into two categories (<8 h or ≥8 h), in accordance with research indicating that Japanese elementary school children’s average sleep duration was 8.5 hours per night.^17^ With respect to food consumption frequency, we asked parents the following question: “Except school lunch, how many times does your child consume fruit and vegetables per week?” There were three response options for the question: almost every day, 3–5 times per week, or 0–2 times per week. The validity of the lifestyle questionnaire was verified in previous studies, in which frequent physical activity was significantly correlated with increased energy expenditure involving physical activity, mean steps taken, and mean activity levels per day measured via the Actiwatch (*P* < 0.05 in a linear trend test).^18^ The correlation coefficient for the association between subjective and objective records of assumed sleep duration was 0.97 (*P* < 0.001).^19^ Body mass index (BMI; weight in kg divided by squared height in m) was calculated using data regarding weight and height measured by trained school nurses at the children’s schools and obtained from the questionnaire. Age- and sex-specific cutoff points that were equivalent to the adult BMI value of 25 for classification as overweight were used to identify children who were overweight.^20^ These cutoff points (19.46 for boys and 19.45 for girls) were developed by the Childhood Obesity Working Group of the International Obesity Task Force, and children with BMI values that exceeded the cutoff points were classed as overweight.

The following questions were used to examine children’s personalities, psychological stress, and unwillingness to attend school: “How often do you feel irritable?” and “How often do you feel like you do not want to go to school?”. There were three response options for the questions: often, sometimes, or rarely. Family environment was measured using questions regarding child-parent interaction (“How often do you usually interact with your parents?”, with three response options: often, sometimes, or rarely), mothers’ employment status (employed full-time, employed part-time, or unemployed), and parental presence (“With whom do you usually eat dinner?”, with three response options: both parents, either parent, or without parents). As almost all fathers were in full-time employment (99.2%), we did not include their data in the analysis.

### Classification of constipation

The definition of constipation is generally vague and ranges from self-reported constipation to the fulfillment of clinical criteria involving bowel movement frequency and symptoms.^1^^,^^2^ In the current study, bowel movement frequency was classified into three categories: at least once daily, once every 2 days, or less frequently than once every 2 days. The occurrence of bowel movements less frequently than once every 2 days was defined as constipation in the current study, because one of the symptoms included in the Roma IV criteria is “two or fewer defecations in the toilet per week”,^2^^,^^21^ which corresponded with our definition.

### Statistical analysis

Distribution and frequency were examined for each category of variables. Chi-square tests were performed to compare the distributions of lifestyle, overweight status, psychological stress, family environment, and bowel movement by sex. Thereafter, logistic regression analysis was performed to evaluate the strength of the associations between these variables and constipation. Boys and girls were then analyzed separately, because sex differences in lifestyles and bowel habits have been reported in previous Japanese studies.^16^^,^^22^^,^^23^ Crude and adjusted odds ratios (ORs) and 95% confidence intervals (CIs) were calculated via binary logistic regression analysis. We adopted a forced-entry method in multivariate models. All analyses were performed using SPSS, version 23.0 J (SPSS, Chicago, IL, USA). A two-tailed *P* value of <0.05 was considered to indicate statistical significance.

## RESULTS

In total, 9,378 participants returned their questionnaires (response rate: 89.8%). Data for 7,998 children (76.6%; 4,157 boys and 3,841 girls) who completed all the questionnaires relevant to our study of constipation were included in the analysis. The average age of participants was 9.76 (standard deviation [SD], 0.29) years. Overall, the prevalence of constipation was 3.9%. In the comparison of boys and girls, the proportion of girls (5.1%) with constipation was higher than that of boys (2.8%). Boys were more likely to be physically active, obese, and to report an unwillingness to attend school, while girls were more likely to watch TV for longer periods, consume fruit and vegetables, and interact with their parents. Almost 40% of mothers were in full-time employment, and approximately 30% were in part-time employment. The proportion of children whose parents were never or rarely present at dinner was greater than 6% (Table 1).

**Table 1.** Characteristics of participants by sex

Total *n* = 7,998		Boys % *n* = 4,157	Girls % *n* = 3,841	*P* value
Skipping breakfast	yes	6.9	6.9	0.960
Physical activity	very often	31.2	24.7	<0.001
	often	46.3	51.5	
	rarely	22.6	23.7	
TV viewing, hours on weekdays				<0.001
	<2	66.5	59.7	
	2 to <3	23.2	28.0	
	≥3	10.3	12.4	
Sleep duration, less than 8 hours		5.4	6.2	0.122
Food frequency (except school lunch)				
Fruit	almost every day	34.5	39.1	<0.001
	3 to 5 times per week	36.2	37.3	
	0 to 2 times per week	29.3	23.7	
Vegetables	almost every day	61.0	63.8	0.001
	3 to 5 times per week	26.1	25.8	
	0 to 2 times per week	12.9	10.4	
Overweight		16.1	13.5	0.001
Psychological stress, family environment				
irritability	often	14.3	15.0	0.520
	sometimes	54.5	54.6	
	rarely	31.2	30.3	
unwillingness to attend school	often	8.0	6.5	0.025
	sometimes	29.2	30.8	
	rarely	62.9	62.6	
interaction with parents	often	21.9	31.1	<0.001
	sometimes	42.7	41.7	
	rarely	35.5	27.1	
mother’s employment	full-time	38.4	40.2	0.029
	part-time	30.6	30.1	
	unemployed	31.0	29.7	
dinner	with both parents	55.4	54.9	0.795
	with either parent	38.5	38.6	
	without parent	6.1	6.5	
Bowel movement	daily or more	78.3	69.0	<0.001
	once every 2 days	19.0	26.1	
	less frequently	2.8	5.1	

Pearson chi-square test.

Table 2 shows the results of the logistic regression analysis of constipation for all participants. In the univariate analysis, girls, physical inactivity, watching TV for long periods, infrequent consumption of fruit and vegetables, being overweight (inversely), frequent irritability, unwillingness to attend school, and infrequent interaction with parents were associated with constipation. In the multivariate analysis, girls (OR 1.97; 95% CI, 1.55–2.51), physical inactivity (OR 1.41; 95% CI, 1.01–1.95), infrequent consumption of fruit (OR 1.68; 95% CI, 1.24–1.94 for 3–5 times per week and OR 1.94; 95% CI, 1.42–2.66 for 0–2 times per week) and vegetables (OR 1.53; 95% CI, 1.17–1.98 for 3–5 times per week and OR 1.46; 95% CI, 1.03–2.05 for 0–2 times per week), being overweight (OR 0.58; 95% CI, 0.40–0.85), frequent irritability (OR 1.76; 95% CI, 1.24–2.50), unwillingness to attend school (OR 1.66; 95% CI, 1.13–2.43), and infrequent interaction with parents (OR 1.55; 95% CI, 1.13–2.13 for sometimes and OR 1.48; 95% CI, 1.06–2.07 for rarely) were significantly associated with constipation. Children whose parents were never or rarely present at dinner were more likely to have constipation compared to those whose parents were present at dinner regularly; however, this difference was not significant. The ORs for the difference in constipation between boys and girls were hardly changed in the multivariate and univariate analyses.

**Table 2.** Overall analyses on constipation (less than once every 2 days)

		Constipation % (95% CI)	Univariate OR (95% CI)	Multivariate OR (95% CI)
Sex	girl/boy	5.1 (4.8–5.6)/2.8 (2.4–3.2)	**1.89 (1.50–2.39)**	**1.97 (1.55–2.51)**
Skipping breakfast	yes/no	4.5 (4.0–5.0)/3.8 (3.3–4.2)	1.19 (0.78–1.80)	0.94 (0.61–1.45)
Physical activity	very often	3.0 (2.6–3.4)	1	1
	often	3.9 (3.5–4.3)	1.29 (0.96–1.73)	1.17 (0.86–1.57)
	rarely	5.0 (4.5–5.5)	**1.68 (1.22–2.31)**	**1.41 (1.01–1.95)**
TV viewing, hours on weekdays				
	<2	3.4 (3.0–3.8)	1	1
	2 to <3	4.2 (3.8–4.6)	1.24 (0.96–1.62)	1.07 (0.82–1.40)
	≥3	5.8 (5.2–6.3)	**1.73 (1.25–2.37)**	1.37 (0.99–1.95)
Sleep duration	<8 h/≥8 h	5.0 (4.5–5.5)/3.8 (3.4–4.2)	1.32 (0.85–2.04)	1.18 (0.75–1.85)
Food frequency (except school lunch)				
Fruit	almost every day	2.5 (2.2–2.8)	1	1
	3 to 5 times per week	4.3 (3.9–4.7)	**1.78 (1.33–2.39)**	**1.68 (1.24–2.26)**
	0 to 2 times per week	5.3 (4.8–5.7)	**2.23 (1.65–3.01)**	**1.94 (1.42–2.66)**
Vegetables	almost every day	3.0 (2.6–3.4)	1	1
	3 to 5 times per week	5.2 (4.7–5.7)	**1.76 (1.37–2.27)**	**1.53 (1.17–1.98)**
	0 to 2 times per week	5.3 (4.8–5.8)	**1.80 (1.30–2.49)**	**1.46 (1.03–2.05)**
Overweight	yes/no	2.6 (2.3–2.9)/4.1 (3.7–4.5)	**0.63 (0.43–0.91)**	**0.58 (0.40–0.85)**
Psychological stress, family environment				
irritability	often	6.6 (6.1–7.1)	**2.21 (1.60–3.06)**	**1.76 (1.24–2.50)**
	sometimes	3.6 (3.2–4.0)	1.18 (0.87–1.60)	1.07 (0.81–1.43)
	rarely	3.1 (2.7–3.5)	1	1
unwillingness to attend school	often	6.9 (6.3–7.5)	**2.06 (1.44–2.93)**	**1.66 (1.13–2.43)**
	sometimes	4.0 (3.6–4.4)	1.17 (0.91–1.51)	1.02 (0.78–1.33)
	rarely	3.5 (3.1–3.9)	1	1
interaction with parents	often	2.7 (2.3–3.1)	1	1
	sometimes	4.3 (3.9–4.7)	**1.61 (1.18–2.20)**	**1.55 (1.13–2.13)**
	rarely	4.3 (3.9–4.7)	**1.63 (1.18–2.26)**	**1.48 (1.06–2.07)**
mother’s employment	full-time	3.9 (3.5–4.3)	1	1
	part-time	4.5 (4.0–5.0)	1.17 (0.90–1.52)	1.22 (0.93–1.59)
	unemployed	3.3 (2.9–3.7)	0.86 (0.65–1.14)	0.92 (0.69–1.23)
dinner	with both parents	3.8 (3.4–4.2)	1	1
	with either parent	3.8 (3.4–4.2)	1.00 (0.79–1.27)	0.97 (0.76–1.24)
	without parent	5.4 (4.9–5.9)	1.44 (0.95–2.19)	1.40 (0.91–2.15)

CI, confidence interval; OR, odds ratio.

Table 3 shows the results of the logistic regression analysis by sex. Boys and girls showed similar associations of constipation with watching TV, food consumption frequency, and being overweight. However, the associations of constipation with physical inactivity (OR 2.28; 95% CI, 1.35–3.85) and unwillingness to attend school (OR 1.90; 95% CI, 1.06–3.42 for often) in boys were stronger compared to those observed in girls. In contrast, the associations between constipation and frequent irritability (OR 1.91; 95% CI, 1.23–2.97 for often) and infrequent interaction with parents (OR 1.74; 95% CI, 1.19–2.55 for sometimes) in girls were stronger compared to those observed in boys.

**Table 3.** Logistic Regression analyses on constipation (less than once every 2 days) by sex

		Boys *n* = 4,157			Girls *n* = 3,841		
	
		Constipation % (95% CI)	Univariate OR (95% CI)	Multivariate OR (95% CI)	Constipation % (95% CI)	Univariate OR (95% CI)	Multivariate OR (95% CI)
Skipping breakfast	no	2.8 (2.3–3.3)	1.01 (0.48–2.08)	0.74 (0.35–1.56)	6.4 (5.6–7.2)	1.30 (0.78–2.18)	1.01 (0.59–1.73)
	yes	2.8 (2.3–3.3)	1	1	5.0 (4.3–5.7)	1	1
Physical activity	very often	1.8 (1.4–2.2)	1	1	4.7 (4.0–5.4)	1	1
	often	2.5 (2.0–3.0)	1.42 (0.86–2.34)	1.32 (0.79–2.19)	5.2 (4.5–5.9)	1.10 (0.77–1.58)	1.06 (0.73–1.53)
	rarely	4.7 (4.1–5.3)	**2.73 (1.63–4.53)**	**2.28 (1.35–3.85)**	5.3 (4.6–6.0)	1.12 (0.74–1.70)	1.00 (0.65–1.54)
TV viewing, hours on weekdays							
	<2	2.4 (1.9–2.9)	1	1	4.7 (4.0–5.4)	1	1
	2 to <3	3.1 (2.6–3.6)	1.31 (0.85–2.03)	1.21 (0.78–1.89)	5.2 (4.5–5.9)	1.12 (0.81–1.57)	1.01 (0.72–1.42)
	≥3	4.4 (3.8–5.0)	**1.90 (1.13–3.20)**	1.58 (0.93–2.69)	6.9 (6.1–7.7)	**1.53 (1.02–2.28)**	1.27 (0.84–1.94)
Sleep duration,	<8 h	3.6 (3.0–4.2)	1.32 (0.63–2.75)	1.19 (0.65–2.53)	6.3 (5.5–7.1)	1.27 (0.74–2.19)	1.15 (0.65–2.01)
	≥8 h	2.7 (2.2–3.2)	1	1	5.0 (4.3–5.7)	1	1
Food frequency (except school lunch)							
Fruit	almost every day	1.7 (1.3–2.1)	1	1	3.2 (2.6–3.8)	1	1
	3 to 5 times per week	2.9 (2.4–3.4)	**1.73 (1.04–2.86)**	1.57 (0.94–2.61)	5.8 (5.1–6.5)	**1.86 (1.30–2.68)**	**1.75 (1.21–2.54)**
	0 to 2 times per week	3.9 (3.3–4.5)	**2.41 (1.47–3.96)**	**1.87 (1.13–3.08)**	7.2 (6.4–8.0)	**2.33 (1.59–3.42)**	**1.99 (1.34–2.97)**
Vegetables	almost every day	2.1 (1.7–2.5)	1	1	4.0 (3.4–4.6)	1	1
	3 to 5 times per week	3.3 (2.8–3.8)	**1.60 (1.04–2.46)**	1.39 (0.90–2.16)	7.4 (6.6–8.2)	**1.89 (1.39–2.58)**	**1.60 (1.16–2.21)**
	0 to 2 times per week	4.9 (4.2–5.6)	**2.39 (1.48–3.85)**	**1.87 (1.13–3.08)**	6.0 (5.2–6.8)	1.52 (0.96–2.41)	1.16 (0.72–1.87)
Overweight	yes	2.2 (1.8–2.6)	0.78 (0.45–1.35)	0.64 (0.36–1.12)	3.1 (2.6–3.6)	**0.56 (0.33–0.94)**	**0.54 (0.32–0.92)**
	no	2.9 (2.4–3.4)	1	1	5.4 (4.7–6.1)	**1**	**1**
Psychological stress, family environment							
irritability	often	4.4 (3.8–5.0)	1.72 (0.92–3.19)	1.55 (0.86–2.78)	8.8 (7.9–9.7)	**2.14 (1.40–3.28)**	**1.91 (1.23–2.97)**
	sometimes	2.7 (2.2–3.2)	1.37 (0.85–2.23)	1.14 (0.72–1.83)	4.6 (3.9–5.3)	1.05 (0.73–1.51)	1.03 (0.72–1.49)
	rarely	2.1 (1.7–2.5)	1	1	4.2 (3.6–4.8)	1	1
unwillingness to attend school	often	5.1 (4.4–5.8)	**2.39 (1.37–4.15)**	**1.90 (1.06–3.42)**	9.2 (8.3–10.1)	**1.99 (1.25–3.18)**	1.54 (0.93–2.56)
	sometimes	3.3 (2.8–3.8)	1.50 (0.99–2.26)	1.29 (0.84–1.97)	4.8 (4.1–5.5)	1.00 (0.72–1.38)	0.89 (0.63–1.24)
	rarely	2.2 (1.8–2.6)	1	1	4.8 (4.1–5.5)	1	1
interaction with parents	often	1.9 (1.5–2.3)	1	1	3.3 (2.7–3.9)	1	1
	sometimes	2.7 (2.2–3.2)	1.46 (0.84–2.56)	1.26 (0.72–2.22)	6.1 (5.3–6.9)	**1.86 (1.28–2.71)**	**1.74 (1.19–2.55)**
	rarely	3.4 (2.8–4.0)	**1.84 (1.06–3.22)**	1.39 (0.79–2.46)	5.7 (5.0–6.4)	**1.74 (1.15–2.62)**	1.51 (0.99–2.29)
mother’s employment	full-time	2.9 (2.4–3.4)	1	1	4.9 (4.2–5.6)	1	1
	part-time	2.9 (2.4–3.4)	1.01 (0.65–1.56)	1.03 (0.66–1.61)	6.2 (5.4–7.0)	1.30 (0.93–1.82)	1.32 (0.94–1.86)
	unemployed	2.5 (2.0–3.0)	0.86 (0.54–1.36)	0.91 (0.57–1.44)	4.3 (3.7–4.9)	0.88 (0.61–1.27)	0.92 (0.63–1.43)
dinner	with both parents	2.8 (2.3–3.3)	1	1	4.9 (4.2–5.6)	1	1
	with either parent	2.5 (2.0–3.0)	0.90 (0.60–1.34)	0.84 (0.56–1.25)	5.2 (4.5–5.9)	1.07 (0.79–1.44)	1.06 (0.78–1.44)
	without parent	4.3 (3.7–4.9)	1.58 (0.82–3.04)	1.52 (0.77–2.97)	6.5 (5.7–7.3)	1.34 (0.78–2.31)	1.33 (0.76–2.31)

CI, confidence interval; OR, odds ratio.

## DISCUSSION

The current results showed that the prevalence of constipation, defined as bowel movements “less frequently than once every 2 days,” was 3.9% in school children aged 9–10 years. Being a girl, physical inactivity, infrequent consumption of fruit and vegetables, psychological stress, and infrequent interaction with parents were associated with childhood constipation. Furthermore, psychological stress and infrequent interaction with parents were as strongly associated with constipation as conventional risk factors, such as infrequent fiber consumption and physical inactivity.

Previous research conducted in Asian countries reported higher prevalence rates for constipation compared to that observed in our study. In a study involving school children in Hong Kong, the prevalence rate was 12.2%.^10^ In another study, 4.4% of Sri Lankan adolescents experienced constipation according to the Roma III Criteria.^24^ In addition, another study showed that 9.3% of Taiwanese children experienced constipation defined according to defecation frequency (<3 times per week).^25^ However, the comparison of prevalence rates is difficult because there is no uniformity regarding the definition of constipation in children.^1^^,^^2^ A review article showed an average self-reported constipation prevalence rate of 20.6%, while the rate calculated according to the Roma III Criteria was 11.0%.^4^ These discrepancies could have occurred because of differences in the assessment of constipation. The Roma criteria were based on both objective data and subjective symptoms; however, the use of only defecation frequency in our study might have led to reduced prevalence.

The results also showed that infrequent consumption of fruit and vegetables was associated with constipation, which was consistent with the findings of previous studies.^25^^–^^27^ A shortage of dietary fiber and fluid intake have been identified as major risk factors for constipation.^25^^,^^28^ In particular, fiber is known to exert a beneficial effect on constipation, because it exerts a fecal bolus mass-incrementing effect, possesses water retention properties, increases colon bacteria and gas production, and accelerates colon transit time.^29^ From our results, having fruit and vegetables almost every day is recommended for child health.

Physical inactivity,^1^^,^^2^^,^^25^ long periods of sedentary behavior,^30^ and short sleep duration^10^ have been reported as lifestyle factors affecting childhood constipation. Tam et al showed that children in Hong Kong having less than 7 hours sleep duration and those who spent 2 hours or longer doing homework reported higher rates of constipation.^10^ While physical inactivity was significantly associated with constipation in the current study, short sleep duration was not (Table 2). There are two possible explanations for this discrepancy. First, the proportion of children with constipation in Hong Kong (12.2%) was higher than observed in the current study (3.9%), and the cutoff points for sleep duration differed (7 hours in Hong Kong and 8 hours in the current study). Second, food consumption frequency and psychological stress were included in the multivariate analysis simultaneously using the forced-entry method only in the current study. Further research is required to examine the association between sleep duration and constipation.

Several previous studies have reported an association between psychological stress and childhood constipation.^7^^,^^31^^,^^32^ For example, Devanarayana et al reported that school- and family-related stressful life evens, such as punishment at school or from parents, bullying, and domestic violence, were associated with higher constipation rates in 2,699 Sri Lankan children. Similarly, psychological stress, including frequent irritability and unwillingness to attend school, was significantly associated with constipation in children in the current study. Stress is believed to modulate the brain-gut axis or hypothalamo-pituitary-adrenal axis and affect gut motility.^2^^,^^33^ Psychological distress has been associated with increased pelvic floor tension and extrinsic gut innervation.^34^^,^^35^ In addition, we demonstrated that psychological stress was as strongly associated with childhood constipation as conventional risk factors, including physical inactivity and infrequent consumption of fruit and vegetables. Therefore, caring for psychological stress could serve as a practical means of preventing childhood constipation.

In our study, factors related to family environment were also assessed. Children who reported infrequent interaction with their parents and those whose parents were rarely present at dinner were more likely to experience constipation compared to other children. Tam et al reported that infrequent parental presence at dinner was significantly associated with children’s constipation.^10^ Our data supported their results. Adequate parent-child interaction and family dinners could be beneficial for children’s bowel movements. We also showed that mothers’ employment status was not associated with constipation. According to the previous studies, children whose mothers were in full-time employment were likely to consume larger amounts of unhealthy food and snacks and smaller amounts of fruit and vegetables, and they were more likely to be obese compared to those with unemployed mothers.^11^^,^^12^^,^^36^ Although the consumption of unhealthy food is considered as a risk factor for constipation,^2^^,^^10^ larger portions, which lead to obesity, could offset the effects of unhealthy food on constipation via a fecal bolus mass-incrementing effect.

Thus far, findings regarding sex differences in the prevalence of constipation have been inconsistent worldwide.^1^^,^^2^ In our study, the finding indicating that girls were more likely to experience constipation compared to boys was consistent with those of other Japanese studies.^23^^,^^37^ Moreover, from stratified analysis by sex, we found that physical inactivity and unwillingness to attend school were strongly associated with constipation in boys, while frequent irritability and infrequent interaction with parents were strongly associated with constipation in girls (Table 3). There are two possible explanations for these sex differences. First, there could have been a greater difference in caloric expenditure between physically active and inactive boys than that observed in girls. Boys are generally more likely to prefer physically active sports, such as baseball, soccer, and other competitive activities, compared to girls, which could have led to a greater difference between active and sedentary boys than in girls. Second, there could be biological differences in gut motility. Gut motility in boys could be more sensitive to exercise, relative to that of girls, while girls’ intestines may be more sensitive to psychological stress via the brain-gut axis compared to those of boys. Emmanuel et al, reported that many patients suffering constipation with psychological distress were women.^35^ This study was the first to analyze factors associated with childhood constipation by sex and demonstrated the importance of sex-specific care for childhood constipation. Further biological research is required to examine the sex difference in childhood constipation.

### Limitations

The large sample size in our study enhanced the validity of the results; however, there were some limitations. First, the study used a cross-sectional design; consequently, we were unable to infer causation. A longitudinal research should be conducted to examine the effects of psychological stress and family environment on childhood constipation. Second, the questionnaire regarding food consumption frequency was not validated, and only a single item pertaining to food consumption frequency was used in the study. In addition, we did not examine food quantities; therefore, the use of detailed questionnaires regarding food consumption is recommended in future research. Third, the questionnaire is subject to recall bias and we did not set a certain period for bowel movement (eg, previous week or month). Measures to minimize recall bias should have been implemented. Fourth, participants in our study were only 9 to 10 years old. It might be difficult to generalize our results to all elementary school children. In addition, other risk factors, such as genetic predisposition and volitional stool retention, were not included. Despite the above limitations, our epidemiological study simultaneously examined factors related to children’s lifestyles, including food consumption frequency, psychological stress, and family environment, and the findings demonstrated the importance of psychological stress and family environment as factors associated with childhood constipation. Therefore, sufficient parent-child interaction and caring for children’s psychological stress could reduce childhood constipation.

### Conclusion

The current results showed that the prevalence rate for childhood constipation was 3.9% in Japanese children and higher among girls. In addition, physical inactivity, infrequent consumption of fruit and vegetables, psychological stress, and infrequent parent-child interaction were associated with childhood constipation. We also showed that psychological stress and infrequent interaction were as strongly associated with constipation as conventional risk factors, such as infrequent fruit and vegetable consumption and physical inactivity. Therefore, clinical doctors, health providers, and parents should prioritize psychological stress and ensure sufficient parent-child interaction in caring childhood constipation.
